# Prevalence and Factors Influencing Post-Operative Complications following Tooth Extraction: A Narrative Review

**DOI:** 10.1155/2024/7712829

**Published:** 2024-05-09

**Authors:** Peter Dignam, Mariam Elshafey, Aparna Jeganathan, Magdalen Foo, Joon Soo Park, Manorika Ratnaweera

**Affiliations:** ^1^UWA Dental School, The University of Western Australia, Nedlands, Australia; ^2^International Research Collaborative—Oral Health and Equity, School of Allied Health, The University of Western Australia, Crawley, Australia; ^3^School of Engineering, Information Technology and Physical Sciences, Federation University Australia, Victoria, Ballarat, Australia

## Abstract

**Background:**

Complications from dental extractions may result in multiple post-operative visits and adversely affect the patient's life. Preventing complications may decrease post-operative morbidity for the individual as well as lower societal costs, such as lost time from work and healthcare costs.

**Objectives:**

This narrative review aims to assess the prevalence and factors influencing post-operative complications following tooth extraction, helping clinicians minimise the risk. *Data Sources*. Cross-sectional studies. *Study Eligibility and Participants*. Patients undergoing dental extractions. Our exclusion criteria included in vitro studies, animal studies, terminally ill patients, and tooth loss not due to dental extraction. Literature was collected from “PubMed” and “Web of Science” through search criteria based on the “PICO” framework. Twenty articles were used to formulate a prevalence table, and 156 articles were included for the factors influencing complications. *Study Appraisal and Synthesis Methods*. This narrative review was reported using the SANRA (a scale for the quality assessment of narrative review articles) checklist. Due to the scope of our narrative review and its associated objectives, the quality of cross-sectional studies (AXIS) will be conducted from the studies outlining the prevalence.

**Results:**

Alveolar osteitis appears to be the most prevalent post-operative complication following tooth extraction. Predisposing factors can be significant in their ability to alter the risk of postoperative complications, and clinicians should provide patient-centred care to mitigate this risk. *Limitations*. Due to the breadth of context, a systematic review was not feasible, as it may have introduced heterogeneity.

**Conclusion:**

This narrative review has highlighted an array of factors which can influence the prevalence of post-operative complications. Future research would benefit from individually reporting post-operative complications, reducing the heterogeneity in definitions of the complications, and including greater detail on the predisposing factors studied.

## 1. Introduction

Dental extractions are well-established interventional procedures performed in many dental practices worldwide. A tooth extraction involves the removal of a tooth or parts thereof, which can range from simple to complex depending on a multitude of factors [1]. Although they are generally considered safe procedures, some complications can arise following a tooth extraction. The incidence of these complications may vary according to specific patient-related, tooth-related, and clinician-related factors [2–9]. Extractions are typically performed as part of the treatment plan for patients with extensive carious lesions or periodontal disease [10]. According to a recent systematic review, tooth decay was the most prevalent reason for tooth extraction (accounting for 36.0%–55.3% of cases) [11].

Patient-related aspects include the patient's health [12], age [2], sex [13–17], smoking habits [18], alcohol consumption [19], medicines used [20], and the quality of dental hygiene practised [21]. Tooth-related variables pertain to details regarding the tooth or teeth being removed and the circumstances surrounding the extraction. Factors such as the indication for tooth extraction [5, 22, 23], the difficulty of the process [24], the arch where the tooth is situated [25, 26], and the exact tooth number are all factors to consider [27]. Clinician-related considerations pertain to the responsibilities of the healthcare practitioner performing the extraction. Factors considered include the clinician's experience [28], surgical techniques [23], anaesthesia type [6], and intra-operative adjuvants used like chlorhexidine gel [9] and platelet-rich fibrin derivatives [29–31]; were pre- and post-operative instructions given like mouth rinses [9, 32], analgesics [33], and antibiotic therapy [34]; and lastly, if a follow-up was conducted [35].

The rate of recovery following dental extractions is mainly dictated by the patient's age, health status, and gingival biotype [36]. Following exodontia, the damaged tissue typically undergoes physiological recovery with accompanying inflammation [36, 37]. This recovery process may manifest as pain, bleeding, bruising, swelling, and trismus. These physiological manifestations may become pathological if the surgical site is infected or other local or systemic factors disrupt the healing process. Therefore, it is important to distinguish manifestations of standard recovery from non-physiological post-operative complications. The pain usually diminishes 7 days following dentoalveolar surgery [38], whereas swelling is 4–5 days [39–41], and oedema is 3–7 days [1]. Bleeding should cease within 8 hr [42]. Trismus can persist up to 7 days [43]. Epithelialisation marks the healing process's conclusion during 14–21 days [44]. However, haematoma does not have a specified timeline other than that it resolves within several days [1]. In addition, complications may arise following infection which can be local or systemic in nature. The presence of fever, malaise, lymph node involvement, or facial cellulitis are all indicative of a systemic rather than local infection [10, 45].

Previous studies demonstrate that certain factors can predispose a patient to a higher risk of post-operative complications and that they can be mitigated through the implementation of specific protocols. This narrative review aims to assess the prevalence and factors influencing post-operative complications following tooth extraction. The implications of these findings provide insight into reviewing and updating the tooth extraction protocols for more favourable patient outcomes.

## 2. Methods

### 2.1. Study Design

**Figure 1:**
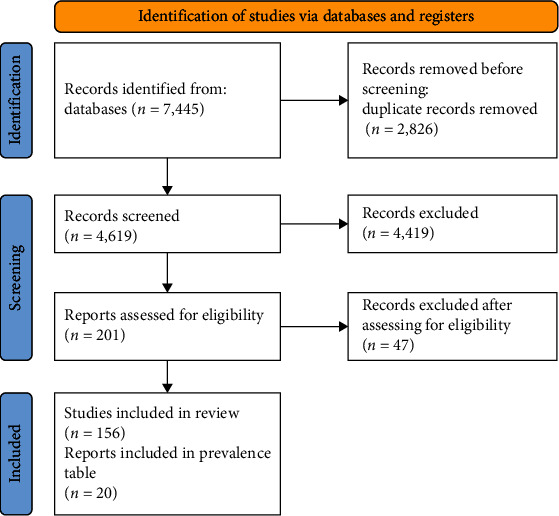
Process of literature collection for prevalence (*n* = 20).

This narrative review was reported using the SANRA (a scale for the quality assessment of narrative review articles) checklist [46]. This review was also registered under the INPLASY database (Number: INPLASY202440028). We have structured our search criteria based on the “PICO” framework. This also underpins our inclusion criteria (*Supplementary 1*). Two researchers simultaneously undertook the research (P.D. and J.S.P.) before the final reviewer (M.R.) verified the search. The population is anyone over 12 years old receiving a dental extraction, the intervention consists of either antibiotics or chlorhexidine, the comparison group is the usual standard of care, and the outcome we are analysing is post-operative complications. The full search terms have been provided in *Supplementary 1*. Our exclusion criteria included in vitro studies, animal studies, terminally ill patients, and tooth loss not due to dental extraction. After searching databases “PubMed” and “Web of Science”, we have generated 7,445 records (Figure 1). After removing duplicate records, we have analysed 201 references for this narrative review, but we have included 20 articles for the prevalence of the post-operative complications [10, 27, 47–64].

### 2.2. Quality and Bias Assessment

Due to the scope of our narrative review, only the studies outlining the prevalence of post-operative complications following tooth extractions will be appraised using the Appraisal Tool for assessing the quality of cross-sectional studies (AXIS) [65]. The AXIS checklist consists of 20 components to assess the quality of the studies included and highlight the potential bias [65]. The majority of the studies had consensus and adhered to the AXIS checklist. However, there were some studies that did not sufficiently describe their methodology or statistical analysis. As well as some studies that did not detail the management of non-responders or limitations (Table 1). The AXIS checklist with the individual studies was provided in *Supplementary 2*.

## 3. Narrative Review

### 3.1. Prevalence of the Post-Operative Complications

The search strategy did not complete the prevalence of the following post-operative complications: facial cellulitis, mental nerve damage, haematoma, and osteonecrosis of the jaw. This is partly due to how some studies combine multiple post-extraction complications into one category. For example, swelling, pain, and trismus were reported together as 0.6% per tooth [49]. Therefore, future research with singularised dependent variables would be ideal to obtain more reliable prevalence scores.

In addition, unifying the terminology used would be beneficial, as “delayed healing” falls short of an informative description of the disease processes experienced by the patient, and reporting on “infection” without defining the disease at hand, such as osteomyelitis, abscess, or facial cellulitis, lacks clarity [51, 63, 66]. This could be achieved by relying on individual post-operative complications with standardised descriptions of their manifestations.

Furthermore, providing the tooth number could enhance the internal validity, as the location does impact the prevalence scores (Table 2). Through this, the external validity would also be strengthened, as the scores could enable clinicians a greater degree of certainty for the risk corresponding to the site-specific surgical treatment at hand. Therefore, the partitioning of some post-operative complications into maxillary and mandibular was performed to incorporate more studies on prevalence, as some studies only described the arch, compared to others which did not specify at all. To explore the impact of tooth type on the prevalence of post-operative complications, a separate search was run on the studies that analysed third molar extractions. However, since not all the other studies specified whether third molars were included amongst the other tooth types, the heading “tooth type not specified” was chosen as opposed to “non-third molar teeth”.

Finally, all prevalence scores reported should have the study's sample size and details considered, for instance, healthy compared to immunocompromised patients. This is because the incorporation of various predisposing factors can, to some degree, impact the patient's recovery outcome. These variables have been categorised into tooth-related, patient-related, and clinician-related variables, which are explored further in this narrative review.

### 3.2. Predisposing Factors: Patient-Related Variables

#### 3.2.1. Health Conditions

The finite list of health conditions complied should be considered alongside any systemic condition which may result in immunosuppression leading to delayed wound healing and increased risk of post-extraction complications [12]. This includes human immunodeficiency virus (HIV), Cushing's syndrome, anaemia, and malnutrition. According to a Cochrane review, physically fit young patients undergoing extraction of their third molars have approximately 10% risk of post-operative infection [67]. However, this increases to approximately 25% if the patient has a compromised immune system.

*(1) Arthritis*. Patients with inflammatory types of arthritis are often treated with disease-modifying anti-rheumatic drugs (DMARDs). The use of DMARDs has been associated with delayed healing due to their immunosuppressive effects and therefore may increase the incidence of medication related osteonecrosis of the jaw (MRONJ) [68]. Likewise, many patients with rheumatoid arthritis (RA) are also on bisphosphonate therapy to manage their risk of osteoporosis [69]. In these patients, there is also an increase in MRONJ prevalence; however, it is not yet obvious whether this is a result of the RA itself or the medications used in its treatment [70, 71].

*(2) Asthma*. Tooth loss in asthmatics using long-term inhaled corticosteroids was reported in association with a decrease in bone mineral density [72]. There is also evidence for increased risk of MRONJ with long-term inhaled corticosteroid use [73]. However, this was studied more broadly and not looked at in the context of MRONJ as a post-extraction complication. Furthermore, long-term corticosteroid therapy has been reported to increase a patient's risk of developing oral infections, namely, candidiasis [74]. However, the effect of this immunosuppression is yet to be substantiated in relation to post-operative complications following tooth extraction.

*(3) Bleeding Disorders*. Patients with bleeding or clotting disorders (e.g., haemophilia or von Willebrand's disease) are at an increased risk of haematoma formation and reactionary bleeding following dental extractions [1, 20, 42, 75]. The severity may vary based on the severity of the bleeding disorder, the size of the surgical site, the number of teeth extracted, the type of teeth extracted, the presence of periodontal inflammation around the extraction site, the presence of vasculopathy, and the level of platelet function [16, 76]. Therefore, it is recommended that patients on anticoagulants have their international normalised ratio (INR) checked 72 hr before exodontia to allow time for dose modification, if necessary, to achieve a safe INR of 2–4 [77].

*(4) Cancer*. Some cancer patients receive radiation therapy as part of their treatment, which may put them at an increased risk of osteoradionecrosis (ORN) [78]. Within radiotherapy-exposed groups, those at the highest risk are those who were treated for oral or oropharyngeal cancer [78]. This is because a high radiation dose of ≥60 Gy to the mandible is one of the strongest risk factors for ORN [60]. This is further increased if the patient is also a smoker [60]. Therefore, radiotherapeutic treatment of oral or oropharyngeal cancer poses a high risk as most head and neck cancers are treated with 70 Gy overlapping or near the mandible [78]. The risk of ORN also increases with time as there was a higher proportion of ORN cases beyond 6 months following initial radiotherapy [79]. Historically, hyperbaric oxygen was used during extraction; the result from the most up-to-date clinical trials (HOPON) has deemed this unnecessary [80]. With that said, systematic reviews and meta-analyses report a lack of clarity for the safest time interval before or after radiation therapy [81–83]. The consensus before is typically providing the wound site as much time as possible to heal, and when extracting after radiation therapy, postponing the extraction for as long as feasible can reduce the risk of ORN. However, caution should always be taken, as dysregulated bone metabolism due to the radiation therapy can persist or even worsen several years after the end of therapy [81].

*(5) Cardiovascular Disease (CVD)*. A population study in Taiwan used the national database to assess the risk of dental infection after extraction in patients with a confirmed ischaemic stroke and myocardial infarction diagnosis [84]. The study reported a 1.15–1.31 times likeliness between these conditions and the prevalence of dental infection [84]. A limitation of this study is that the researchers were unable to determine disease severity because the International Classification of Disease (ICD) coding system was used. Separately, it has been reported that patients with a history of infective endocarditis are more susceptible to post-extraction infections [85, 86]. Antibiotic prophylaxis can be indicated to prevent relapse of infective endocarditis; however, there is limited evidence that this prevents localised infection.

*(6) Chronic Kidney Disease (CKD)*. It has been reported that patients with chronic kidney disease have a proclivity for infection [87]. Therefore, a Cochrane review reported a likely benefit from prophylactic antibiotic therapy for patients with a higher risk of infection [67]. This is attributed to delayed wound healing and prolonged bleeding after tooth extraction, which may be related to the patient's immunosuppressant medication alongside reduced kidney function [88]. However, a pilot study reported that patients with chronic kidney failure that were not given prophylactic antibiotics experienced prolonged bleeding but not an increased risk of post-operative infection [89]. A limitation to this study is that only simple extractions were investigated [89]. To reduce the risk of PEB, it is recommended that tooth extractions are booked 1 day after dialysis, as this is when the anticoagulant agent's concentration is the lowest [87].

*(7) Chronic Obstructive Pulmonary Disease (COPD)*. The correlation between periodontal disease and COPD has been previously established [90, 91]. Additionally, a prospective study of 20 individuals with COPD reported a greater number of dental infections and tooth extractions amongst this patient cohort [92]. However, there were no studies found to suggest an increased prevalence of post-operative complications following tooth extraction for patients with COPD.

*(8) Diabetes Mellitus*. Many health conditions, such as diabetes mellitus, can influence wound healing largely due to reduced angiogenesis and decreased collagen synthesis [12, 31, 93]. Studies have demonstrated that this impaired ability to heal is more typical of people with uncontrolled diabetes [94–97]. However, not all studies support glycaemic control as a risk factor for post-operative complications following tooth extraction [98–100].

*(9) Osteoporosis*. Most patients with osteoporosis are on anti-resorptive drugs (ARDs), such as bisphosphonates, to manage their condition [101]. ARDs increase the risk of MRONJ through impaired wound healing via inhibition of osteoblasts and osteoclasts, leading to suppression of bone remodelling [102].The duration of antiresorptive medication use is the most important factor in determining the risk of osteoporosis patients developing MRONJ [103, 104]. The risk is up to four times higher for patients with an at least 3–4-year history of antiresorptive drug use [101, 104, 105]. Moreover, there is also a significantly higher number of MRONJ cases post-extraction for patients using injectable antiresorptive drugs compared to those using oral forms (*P*=0.036) [106].

#### 3.2.2. Age

Older patients with more comorbidities have an increased risk of experiencing post-operative complications [2]. Age as a sole factor can also increase an individual's risk of a post-operative complication following tooth extraction [107]. A cohort study investigating 120 patients (*n* = 550, teeth extracted) reported a weak but significant increase in tooth loss for older patients (*P*  < 0.001) [108]. Therefore, not only do older patients have a high rate of tooth extraction, but they also have higher rates of post-extraction complications [107]. This can be explained by the body's reduced ability to heal throughout life [109].

#### 3.2.3. Sex

Multiple studies have reported that the rate of oral mucosa healing is significantly slower in females than in males [13–17]. Meanwhile, other studies have reported no sex difference [110, 111]. In addition, orofacial pain following dental treatment was found to be more common amongst females [112]. However, a limitation of this study is that it did not isolate post-extraction orofacial pain from other dental sources of iatrogenic orofacial pain. Separately, the risk of post-extraction alveolitis and infection was reported as being more common among females [113].

#### 3.2.4. Smoking Habits

Smoking should be avoided after a tooth extraction, as it can disrupt the clot formation process necessary for healing [18]. Links between smoking and higher levels of post-operative pain compared to non-smokers have also been made (*P* < 0.0001) [114]. Additionally, smokers were more likely to experience facial swelling (*P*=0.04) and post-operative infection (*P* > 0.05) [114]. However, they were less likely to experience PEB [114]. A systematic review investigating 11 studies reported tobacco smokers have over a threefold increase in the odds of developing alveolar osteitis after tooth extraction [115]. This was corroborated by another study that found the incidence of developing dry socket was around 13.2% in smokers, compared to 3.8% in non-smokers [3]. The increase in detrimental risks following dental treatment applies to both e-cigarette and conventional cigarette smokers [115]. However, the former was affected to a lesser extent according to a systematic review.

#### 3.2.5. Alcohol Consumption

Alcoholism can reduce a patient's healing capacity after oral surgery [116]. However, the chance of haemorrhage for dental extractions is considered low as no vital organs are involved, there is limited dissection, and local haemostatic measures are often adequate [19]. In addition, chronic alcoholism can cause thrombocytopenia and thrombocytopathy [19], which may predispose patients to an increased incidence of infection [4, 83].

#### 3.2.6. Medications

The three main drug classes this review collated were anticoagulants, oral contraceptives, and immunosuppressants. A Cochrane review reported patients on continuous oral anticoagulant therapy have a greater risk of bleeding complications during and after dental extractions [20]. This review highlighted that the use of antifibrinolytic agents, namely, locally applied tranexamic acid solution, may lead to a 25% reduction in PEB [20]. However, the heterogeneity between the studies makes it difficult to draw this as an absolute conclusion. Oral contraceptives are believed to increase fibrinolysis and hence delay healing [117]. A meta-analysis reported that females that use oral contraceptives were almost twice as likely to develop alveolar osteitis following a third molar extraction, compared to those that do not [117]. However, it has also been reported that there is no significant relation between the use of oral contraceptives and alveolitis [113]. Immunosuppression negatively influences wound healing [67]. Therefore, there is an increased risk of delayed wound healing and MRONJ in patients using prolonged corticosteroid therapy, biological agents, or DMARDs [68]. Post-operative complications are not necessarily caused by these drugs following tooth extraction, but they act as risk factors which should be taken into consideration when treatment planning and prioritising post-operative reviews [68]. For instance, biological DMARDs, largely used to treat patients with autoimmune disease, have been associated with maxillary and mandibular MRONJ after exodontia, and the importance of wound closure and antibiotic therapy is evidenced for these patients [118, 119].

#### 3.2.7. Oral Hygiene

The level of oral hygiene at the time of extraction as well as during the post-operative healing period is correlated with some post-operative complications [21]. Poor oral hygiene was correlated with higher pain levels especially in the first 48 hr post-operatively [120]. As a result, there was also an increased number of analgesics used by this patient cohort, yet they still had higher pain scores (*P*=0.048) [120]. This was further corroborated by another study that found pain levels were 2.98 times higher in patients with poorer oral hygiene (*P*  < 0.05) [21]. It is hypothesised that a higher bacterial load leads to higher production of microbial toxins which may in turn trigger inflammatory pathways leading to increased pain levels [120]. Despite this, no significant correlation was found between oral hygiene levels and levels of inflammation, trismus, or swelling post-extractions [21, 120]. There is, however, a correlation between the incidence of alveolar osteitis and oral hygiene, with a significance of *P*  < 0.05 for fair and *P*  < 0.035 for poor oral hygiene [121]. Another study reported the risk of alveolar osteitis was 3.65 times greater in patients with poor oral hygiene [5].

### 3.3. Predisposing Factors: Tooth-Related Variables

#### 3.3.1. Indication for Tooth Extraction

It has been reported that the presence of an existing infection before the extraction can increase the risk of post-extraction complications [5, 22, 23]. For instance, pericoronitis can increase the likelihood of post-operative sequalae [10].

When a tooth is indicated for extraction due to impaction, distally angulated mandibular third molars have been reported as a risk factor for post-operative pain, swelling, and trismus (*P* ≤ 0.05) [23]. In terms of distally angulated maxillary third molars, another study reported this presentation had the greatest risk of oral–antral communication [48].

Additionally, a higher risk of MRONJ was found following extractions that were done due to vertical root fracture, periodontal disease, or periapical pathosis in comparison to the other investigated indications in a cohort study of 93 patients using bone-modifying agents (*P*=0.01) [122]. Furthermore, another study reported that the likelihood of MRONJ after tooth extraction significantly increased by 2.6 times when the patient was diagnosed with periodontal disease [123]. Therefore, patients taking bisphosphonates should have their periodontal diseases managed prior to dental extractions [123]. For patients that have already begun using bone-modifying agents, extractions should be carried out sooner rather than later to avoid further increasing their risk of MRONJ [124].

It is unclear whether periodontal disease influences the risk of post-operative complications. A retrospective analysis reported that teeth with marginal periodontitis typically required an easier extraction (9%, *n* = 723), compared to teeth with periapical periodontitis which were deemed more complex (34%, *n* = 5,170) (*P* < 0.001) [125]. This could have an implication for the association between extraction complexity and duration with post-operative complications [125].

There is limited evidence about the correlation between teeth extracted due to extensive decay and the risk of developing post-extraction complications. One study reported a higher incidence of these complications when extracting carious third molars with a potential jaw fracture [126].

#### 3.3.2. Extraction Complexity and Procedure Duration

There is a positive correlation between the complexity of an extraction and the incidence of post-extraction complications [24]. In a study of mandibular third molars, complex extractions had a statistically significant higher incidence of pain, oedema, trismus, and paraesthesia 1-day post-operatively potential as a result of greater inflammation [8].

For third molar extractions, the position of the tooth is the main method for classifying surgical difficulty. The two main systems used are Pell and Gregory or Winter's classification system [24]. Each tooth is categorised as either being simple, advanced, or complex. However, the validity of these classification systems in predicting the actual surgical difficulty has been questioned due to omitting patient factors and surgical complexity [127]. The same study found that increased patient age and weight lead to more complex extractions due variances in bone density, bone quality, root morphology, and difficulty in surgical access [127]. Additionally, for non-third molar teeth, factors that may increase the complexity of an extraction include the need for tooth sectioning, flap incision, or bone removal [10].

It has also been noted that more complex procedures are typically associated with longer procedure durations [10]. As such, a correlation has also been reported between longer procedures and the increased incidence of post-extraction complications. A prospective study on 532 patients reported the incidence of post-extraction complications was 3.08% for procedures that lasted 0–15 min, 3.44% for those lasting 16–30 min, and 5.2% if the procedure exceeded 30 min [128]. However, it is difficult to determine whether it was the procedure duration alone or the increased procedure complexity that contributed to the increase in post-extraction complications.

#### 3.3.3. Tooth Arch

Post-operative complications are typically more prevalent in the mandible than the maxilla [25, 26]. A study of third molars found that 80.6% of the post-operative complications experienced had occurred in the mandibular arch [49]. The main reason for this is that the mandible has less spongy bone and thicker cortical plates than the maxilla [25, 26]. Therefore, the tooth sockets expand less rapidly, and so greater pressure must be applied during mandibular exodontia. This increases the operative duration and complexity, thus increasing the risk of post-extraction complications [25, 26]. The increased force applied to the depressed mandible over the maxilla also confers a higher risk of trismus and temporomandibular joint injuries. Furthermore, the reduced vascularisation of the mandible also leads to slower healing which increases the risk of developing post-extraction complications [25, 26].

#### 3.3.4. Tooth Number

Extraction of third molars is typically associated with a greater prevalence of post-operative complications than any other tooth type [27]. Extraction of third molars as opposed to any other tooth increases the risk of post-operative complications (*P*=0.024) especially for infection (*P*  < 0.001) and nerve damage (*P*=0.027) [27]. This is likely because the procedure is typically more complex and generally takes longer [24]. Complicating factors may include less access to the operative field, tooth impaction, and proximity of tooth roots to the inferior alveolar nerve [129]. In addition, the risk of alveolar osteitis increases to upward of 30% when undertaking third molar surgical extractions [9].

### 3.4. Predisposing Factors: Clinician-Related Variables

#### 3.4.1. Level of Experience

Experienced general dental practitioners and specialist dentists would be expected to perform extractions more easily than less experienced graduates and dental students. One study investigated confidence levels across two dental schools using a five-point scale, reporting neither average surpassed neutral [28]. In addition, the prevalence of postoperative infection after tooth extraction was recorded at 22 infections when the extraction was performed by a dental student, compared to three infections for those performed by a dental surgeon [10].

#### 3.4.2. Surgical Removal Techniques

Post-operative complications have been associated with the use of flaps with vertical incision and removal of bone (*P* ≤ 0.05) [23]. Therefore, when appropriate, a minimally invasive approach should be opted for. A meta-analysis containing nine eligible studies reported the relative risk of lingual nerve injury following three different surgical techniques for mandibular third molar extraction, all of which resulted in a low incidence of lingual nerve impairment [130]. It was demonstrated that a buccal approach with lingual flap retraction compared to a buccal approach without lingual flap retraction had a relative risk of 4.80 (*P*  < 0.00001) [130]. Furthermore, a Cochrane review reported that compared to envelope flaps, triangular flaps resulted in a 71% reduction in alveolar osteitis and a reduction in pain [131]. However, residual swelling was lower when an envelope flap was used [131].

#### 3.4.3. Anaesthesia

This narrative review found unequivocal themes, with some studies suggesting no significant correlation, while others state there is a higher incidence of post-operative complications for extractions performed under general anaesthesia (GA) compared to those performed under local anaesthesia (LA) [6]. However, it is more likely that these noted differences are a result of the longer operation times and increased extraction complexity rather than as a direct result of the anaesthetic modality used [6, 132, 133].

One study found a statistically significant increase in the frequency of alveolar osteitis cases associated with extractions under LA over those under GA [6]. However, the reason was not given, and other studies have shown no significant correlation between the two [63, 134, 135]. In the past, it was hypothesised that excessive use of vasoconstrictors may cause local ischaemia and increase the risk of alveolar osteitis [136]. Since then, several studies have shown no significant correlation [134–136]. Similarly, no difference was found between the use of regional blocks versus infiltrations and the incidence of alveolar osteitis [136].

A higher risk of lingual nerve injury for surgeries performed under GA has been reported (*P*=0.02) [63]. Another study showed that there was a 16.49 times higher risk of inferior alveolar nerve injury under GA when compared to LA (*P*  < 0.001) [137]. It was hypothesised that this was because more of the teeth extracted under GA had an acute pathology with highly anxious patients [137]. In terms of conscious sedation, it has been reported to be beneficial in lowering patient anxiety and reducing post-surgical pain when combined with LA, compared to LA alone [138, 139].

#### 3.4.4. Chlorhexidine (CHX) Mouth Rinse

The main mouth rinse investigated in the literature is chlorhexidine (CHX) antiseptic mouth rinse, which can be administered either pre-operatively, post-operatively, or both pre- and post-operatively. Post-operative administration was determined adequate and more feasible than both pre- and post-operative administrations in reducing the incidence of alveolar osteitis after the surgical removal of third molars [32]. A general consensus on the administration of the CHX mouth rinse followed a regimen of swishing 15 mL for 30 s twice a day 1 week before and/or 1 week after surgery [32]. Alternatively, a single dose of 15 mL of CHX mouth rinse for 30 s immediately prior to the mandibular third molar extraction also showed a reduction in the incidence of alveolar osteitis (*P*  < 0.05) [140]. However, this was only observed when the mouth rinse was used in combination with a *β*-lactamase-containing antibiotic, but no antibiotic control group was present in this study. A Cochrane review demonstrated that both pre- and post-operative CHX mouth rinsing (0.12% and 0.2% concentrations) reduced the risk of alveolar osteitis by 42% (*P*  < 0.001) [9]. Furthermore, this Cochrane review has been updated to report that CHX mouth rinses (0.12% and 0.2%) both before and 24 hr after an extraction also reduced the risk of alveolar osteitis (*P*  < 0.00001).

### 3.5. Analgesics

According to a Cochrane review, ibuprofen 400 mg combined with paracetamol (acetaminophen) 1,000 mg significantly reduced a patient's pain with a risk ratio of at least 50% pain relief at 6 hr for pain relief after surgical removal of lower wisdom teeth [33]. The combined therapy was better than monotherapy. When used as a monotherapy, ibuprofen 400 mg provides more pain relief than 1,000 mg paracetamol.

#### 3.5.1. Antibiotics

The prophylactic use of antibiotics is generally not indicated for extractions unless the patient has experienced numerous accounts of alveolar osteitis, is at an increased risk of infective endocarditis, or is immunocompromised [34].

A recent Cochrane review, which included 23 randomised trials involving the surgical removal of third molars, reached a conclusion based on moderate-quality evidence that antibiotics may reduce the risk of alveolar osteitis by 38% (*P*=0.03) [67]. However, this should be weighed up against the increased risk of adverse events associated with antibiotic use such as nausea, vomiting, and diarrhoea (*P*=0.02) [67]. The review also states that if antibiotics are not indicated prophylactically, they should be reserved for post-operative prescription following signs of infection, due to the increase in antibiotic resistance [67]. Dentists account for approximately 10% of the antibiotics prescribed; therefore, it is imperative to practise antibiotic stewardship [141]. The typical prophylactic pre-operative regimen consists of 2 g of amoxicillin 1 hr before the procedure, unless the patient has a penicillin allergy or is on long-term penicillin therapy, in which case either 2 g of cefalexin or 600 mg of clindamycin is used instead [142]. Prophylactic usage of post-operative antibiotics varies more in the literature, such as amoxicillin/clavulanic acid 500/125 mg three times a day for 4 days [143], amoxicillin 500 mg every 8 hr for 5 days with metronidazole 400 mg every 8 hr for 5 days [144], or amoxicillin 500 mg taken three times daily for 5 days after surgery [145].

#### 3.5.2. Chlorhexidine (CHX) Gel

A Cochrane review reported the intra-socket use of 0.2% CHX gel after dental extraction significantly reduced the odds of developing alveolar osteitis (*P*=0.0008) [9]. Moreover, a meta-analysis of 11 studies showed that 0.2% CHX gel significantly reduced the post-operative complications of third molar extractions by 62% (*P*  < 0.00001) [146]. Likewise, another meta-analysis of 52 studies also supported this finding (*P* < 0.00001) [147]. Furthermore, another study found that there was significantly faster wound closure after using 0.2% CHX gel (*P*  < 0.05) [148]. However, this study focused only on the early stages of healing so it may not be applicable to those suffering from chronic wound healing [148]. A randomised controlled trial comparing the impact of 0.2% CHX intra-socket gel to 0.12% CHX mouth rinse during the first week post-operatively reported the gel group had a lower incidence of alveolar osteitis at 7.5% compared to 25% in the mouth rinse group (*P*  < 0.05) [149].

#### 3.5.3. Platelet-Rich Fibrin Derivatives

Systematic reviews and meta-analyses have reported topical platelet derivatives, such as platelet-rich fibrin (PRF) and plasma-rich in growth factors (PRGF), can improve wound healing and increase bone density, stimulating regeneration of the soft tissues and bone [29–31]. One meta-analysis showed that platelet derivatives do not prevent post-operative sequalae, as from the 10 randomised controlled trials they could only reduce sequalae, such as pain (*P*  < 0.05) [30]. Another meta-analysis has shown after the exodontia of impacted third molars, the use of PRF reduces swelling, pain, and the risk of alveolar osteitis [150]. In addition, PRF membranes applied after exodontia in patients undergoing treatment for osteoporosis may reduce the risk of complications, such as MRONJ [151]. PRF is not used as a membrane but rather for socket preservation and to aid healing. However, a Cochrane review has found no evidence to refute or confirm a benefit of PRF or PRGF inserted into the post-extraction alveolus for the prevention of MRONJ [152].

#### 3.5.4. Follow-Up

Establishing a follow-up appointment for patients' post-extraction can be a contentious topic with some clinicians feeling that it wastes clinical time and provides questionable benefits for patients [35]. It was reported that less than half of the patients who believed they did not have adequate healing 24–72 hr after surgery felt the need to visit their dental practitioner [153]. Therefore, a lack of following up may potentially hinder early detection and treatment of post-operative complications [153]. Phone follow-ups decrease this by providing a convenient and low-cost option for patients, as 95% of patients preferred not to come back for follow-up appointments in person unless suture removal was required [153]. However, tele-dentistry is not the complete solution, as there are limitations associated with patient self-reporting and clinician detection without visual diagnosis [153, 154].

### 3.6. Strengths and Limitations

Due to the breadth of context, a systematic review was not feasible, as it may have introduced heterogeneity. Furthermore, there should be an adequate number of papers to account for the need for a systematic review, but based on our research, it was difficult to find such studies that satisfied all the aspects of this review. In the future, this might be an avenue we can explore. However, we have utilised the SANRA checklist to ensure transparency [46]. Therein, as this is a narrative review, only cross-sectional studies highlighting the prevalence were assessed for their quality (AXIS). The validity of the papers reviewed were typically of high calibre; however, a justification of the sample size and addressment of non-responders were lacking. Within the review of the prevalence, only one study was able to address the non-response bias indicating that the majority of the studies falls under the moderate to high risk of bias [62]. The results appeared to be of high quality, although the limitations across the papers could have been divulged more thoroughly [10, 47, 48, 55, 59, 61].

### 3.7. Implications to Future Research

Further systematic reviews on the predisposing factors are required to ascertain more reliable and accurate conclusions. Moreover, analysing specific complications in conjunction with the numerous predisposing factors would add to the existing knowledge. Due to the lack of evidence available in the literature, future studies could investigate complication rates within a tertiary hospital setting to achieve this, and more research on the duration of physiological complications following tooth extraction would add to an apparent gap in the literature and consolidate what was provided in this narrative review. Furthermore, it has been demonstrated that recently introduced treatments such as ozone [155] and photobiomodulation [156] have a significant influence on oral environment. Therefore, it would be interesting to test in future reports the efficacy of these compounds and techniques also on post-operative complications following tooth extraction.

In addition, our study creates a baseline to assess how to avoid these complications and how to control all these factors to reduce morbidity. Lastly, this narrative review revealed an opportunity for a Delphi consensus study to determine standardised terminology and definitions of the post-extraction complications experienced.

## 4. Conclusion

The extent and level of studies focusing on alveolar osteitis highlight how it is perhaps the most prevalent post-operative complication following tooth extraction. The numerous predisposing factors reported are important, as they indicate significant risk for the patient's recovery. A patient-centred approach should be taken, as specific factors can pose elevated risk for specific post-extraction complications. It is a clinician's responsibility to use this information to identify the potential risks, discuss these with the patient, and mitigate all unnecessary harms. As this is a narrative review, no factor can be held more significant than another. Nevertheless, it is important to consider all factors when determining a patient's individual risk.

## Figures and Tables

**Table 1:** Quality assessment of cross-sectional literature collected for prevalence (*n* = 20).

Questions	*Y*	*N*	*U*	*N* (A)
Introduction				
Were the aims/objectives of the study clear?	18	1	1	0
Methods				
Was the study design appropriate for the stated aim(s)?	19	0	1	0
Was the sample size justified?	0	18	1	1
Was the target/reference population clearly defined? (Is it clear who the research was about?)	20	0	0	0
Was the sample frame taken from an appropriate population base so that it closely represented the target/reference population under investigation?	18	0	1	1
Was the selection process likely to select subjects/participants that were representative of the target/reference population under investigation?	17	0	2	1
Were measures undertaken to address and categorise non-responders?	11	7	1	1
Were the risk factor and outcome variables measured appropriate to the aims of the study?	19	1	0	0
Were the risk factor and outcome variables measured correctly using instruments/measurements that had been trialled, piloted, or published previously?	17	0	3	0
Is it clear what was used to determine statistical significance and/or precision estimates (e.g., *p* values and confidence intervals)?	15	5	0	0
Were the methods (including statistical methods) sufficiently described to enable them to be repeated?	14	4	1	0
Results				
Were the basic data adequately described?	20	0	0	0
Does the response rate raise concerns about non-response bias?	1	19	0	0
If appropriate, was information about non-responders described?	1	0	0	19
Were the results internally consistent?	19	0	1	0
Were the results presented for all the analyses described in the methods?	17	1	2	0
Discussion				
Were the authors' discussions and conclusions justified by the results?	20	0	0	0
Were the limitations of the study discussed?	14	6	0	0
Other				
Were there any funding sources or conflicts of interest that may affect the authors' interpretation of the results?	15	5	0	0
Was ethical approval or consent of participants attained?	15	5	0	0

**Table 2:** Prevalence of post-operative complications in third molar and non-third molar teeth.

Post-operative complication	Tooth type not specified		Third molar teeth	
Alveolar osteitis	0.78%−39.12% [27, 51, 58, 59]		0.19%–12.7% [27, 54]	
	Maxillary = 1.21% [59]	Mandibular = 2.69% [59]	Maxillary = 0.38% [48]	Mandibular = 2.7%–4.2% [47, 50, 63]
Osteomyelitis	0.7% [27]		0.32%−0.37% [27, 47]	
Abscess	0.11% [10]		0.32%–1.25% [47, 48]	
Facial cellulitis	No reports found		0.08% [64]	
Pain	0.86%−6.76% [10, 27, 58]		0.3%–1.6% [27, 50, 52]	
Inferior alveolar nerve damage	0.06% [27]		0.23%–8.4% [47, 52, 54, 63]	
Lingual nerve damage	0.08% [27]		0.2%−5.7% [47, 49, 50, 54]	
Mental nerve damage	No reports found		No reports found	
Haemorrhage	0.55%−13.51% [27, 58]		0.6%–0.7% [27, 52]	
			Maxillary = 0.064% [48]	Mandibular = 0.3%–0.4% [47, 50]
Haematoma	No reports found		0.08% [47]	
Trismus	14.85% [58]		0.3%−0.41% [51, 53]	
Swelling/oedema	6.75% [58]		0.06% [52]	
Osteonecrosis of the jaw ^*∗*^	1.9%−3.44% [55, 57]		No reports found	
Osteoradionecrosis ^*∗∗*^	8.5%−19.8% [56, 60]		28.6% [62]	
	Maxillary = 2.2%−9.1% [56, 60, 61]	Mandibular = 9.6%–17.6% [56, 60, 61]		
Oral–antral communication	0.94% [27]		2.4% [48]	

^*∗*^Of patients on oral or intravenous bisphosphonates.  ^*∗∗*^Of patients with a history of head and neck radiotherapy.

## Data Availability

The data supporting this research article are available from the first or corresponding author on request.

## Supplementary Materials

Supplementary 1 PICO-generated search terms and MeSH terms.

Supplementary 2 AXIS checklist with individual studies.
