# Supplementary material for: Prevalence and Factors Influencing Post-Operative Complications following Tooth Extraction: A Narrative Review
Source: Int J Dent. 2024 May 9;2024:7712829. doi: 10.1155/2024/7712829 (PMC11098612; doi:10.1155/2024/7712829)
Supplement: Supplementary 1 — PICO-generated search terms and MeSH terms. [file 7712829.f1.docx]

# Supplement

## Supplement 1: PICO generated search terms

Description - We identified studies on dental surgery candidates using the Population, Intervention, Comparator, Outcome (PICO) framework, focusing on dental extraction interventions and post-surgery outcomes. Exclusions were research on animals, children, and *in vitro* models.

Search strategy:

- Searching ‘PubMed’ and ‘Web of Science’.
- The following terms were searched in the title, abstract, and keywords.
- The Boolean operator was applied for the population, intervention, and outcome search threads.
- The exclusion criteria were studies done in animals, children, and in vitro models.

| Population | - “pre-op* variable” OR “risk” OR “factor” OR “sex” OR “gender” OR “female” OR “male” OR “intersex” OR “non-binary” OR “ethnicity” OR “age” OR “elderly” OR “health*” OR “medically fit” OR “medically compromised” OR “disorder” OR “disease” OR “condition” OR “cardiovascular disease” OR “CVD” OR “osteoporo*” OR “bisphosphonate” OR “irradiat*” OR “radiotherapy” OR “cancer” OR “immunosuppress*” OR “rheumatoid arthritis” OR “RA” OR “anticoagulant” OR “chronic kidney failure” OR “diabet*” OR “glycaemic control” OR “medic*” OR “pharmac*” OR “drug” OR “smoking” OR “tobacco” OR “cigarette” OR “alcohol” OR “oral contracepti*” OR “simple” OR “complex” OR “surgical” OR “non-surgical” OR “tooth type” OR “third molar” OR “wisdom t**th” OR “arch” OR “maxilla*” OR “mandib*” OR “anaesthesia” OR “conscious sedation” OR “oral hygiene” OR “prophylaxis” OR “prevent*” OR “interven*” OR “mouth wash” OR “mouth rinse” OR “chlorhexidine” OR “CHX” OR “analgesics” OR “ibuprofen” OR “paracetamol” OR “platelet-rich fibrin derivatives” OR “antibiotic” OR “amoxicillin” OR “tertiary” OR “student” OR “general dentist” OR “specialist” OR “surgeon” OR “follow-up” OR “systemic” OR “immunocompetent” OR “DMARD” OR “COPD” OR “chronic obstructive pulmonary$” OR “anti-coagulant” OR “anti-platelet” OR “autoimmune disease” OR |
| --- | --- |
| Intervention | **Search 1:**   - “exodonti*”   **Search 2:**   - “removal” OR “extract*” OR “surgery”   AND   - “t**th” OR “dental” OR “third molar” OR “wisdom t**th” OR “surgical” OR “dentoalveolar” OR |
| Comparison | No comparison. |
| Outcome | - “safety” OR “danger” OR “outcome” OR “morbidity” OR “healing” OR “recovery” OR “wound” OR “defect” OR “sequalae” OR “complication” OR “impair*” OR “alveolar osteitis” OR “dry socket” OR “osteomyelitis” OR “abscess” OR “swelling” OR “o*dema” OR “cellulitis” OR “deep fascial space infection” OR “pain” OR “nerve” OR “neurosensory” “*sthesia” OR “bleeding” OR “haemorrhage” OR “haematoma” OR “trismus” OR “osteonecrosis” OR “*RONJ” OR “osteoradionecrosis” OR “ORN*” OR *oral antral communication” OR “OAC” OR “oral antral fistula” OR “OAF” |

## Supplement 2: MeSH Terms

Description - The list encompasses MeSH terms related to dental surgery, focusing on conditions, causes, demographics, and post-operative effects. These terms aid in systematic research for related studies for the narrative reviews (https://www.ncbi.nlm.nih.gov/mesh/).

| **MeSH Terms by Alphabetical Order** |
| --- |
| - Abscess - Adult - Adverse effects - Aged - Alveolar osteitis - Antibiotics - Bruise - Chlorhexidine gel - Chlorhexidine mouth rinse - Dental audit - Dry socket - Exodontia - Extraction - Facial cellulitis - Facial paraesthesia - Female - Geriatric - Haematoma - Haemorrhage - Hematoma - Hemorrhage - Humans - Immunocompromised host - Incidence - Infection - Lingual nerve injury - Male - Medical history taking - Medication - Molar - Nerve impairment - Numerical data - Oedema - Oral antral communication - Osteomyelitis - Osteonecrosis of the jaw - Osteoradionecrosis of the jaw - Pain, Post-operative - Post-operative complications - Predisposing factors - Prophylaxis - Retrospective studies - Risk factors - Schools, Dental - Statistics - Surgery - Swelling - Third molar - Tooth extraction - Trismus - Young adult |
