# Supplementary material for: Prevalence and Factors Influencing Post-Operative Complications following Tooth Extraction: A Narrative Review
Source: Int J Dent. 2024 May 9;2024:7712829. doi: 10.1155/2024/7712829 (PMC11098612; doi:10.1155/2024/7712829)
Supplement: Supplementary 2 — AXIS checklist with individual studies. [file 7712829.f2.pdf]

Supplement 3 - Assessment of the individual studies included in the systematic review according to the Appraisal tool for Cross-Sectional Studies (AXIS)

**Questions**	Sigron 2014	Pourmand 2014	Sayed 2019	Yamada 2022	Chen 2021	Miclotte 2018	Bui 2003	Momin 2018	Haug 2005	Jeong 2017	Khoo 2021	Otto 2015	Saravanan 2021	Yue Yi 2021	Upadhyaya 2010	Möring 2022	Bodem 2015	Oh 2004	Osunde 2014	Yoshii 2001
Introduction																				
Were the aims/objectives of the study clear?	Y	Y	Y	U	Y	Y	Y	Y	N	Y	Y	Y	Y	Y	Y	Y	Y	Y	Y	Y
									Not very clear											
Methods																				
Was the study design appropriate for the stated aim(s)?	U	Y	Y	Y	Y	Y	Y	Y	Y	Y	Y	Y	Y	Y	Y	Y	Y	Y	Y	Y
Was the sample size justified?	N	N	N	N	N/A	N	N	N	U	N	N	N	N	N	N	N	N	N	N	N
Was the target/reference population clearly defined? (Is it clear who the research was about?)	Y	Y	Y	Y	Y	Y	Y	Y	Y	Y	Y	Y	Y	Y	Y	Y	Y	Y	Y	Y
Was the sample frame taken from an appropriate population base so that it closely represented the target/reference population under investigation?	Y	Y	Y	Y	N/A	Y	Y	Y	Y	U	Y	Y	Y	Y	Y	Y	Y	Y	Y	Y
Was the selection process likely to select subjects/participants that were representative of the target/reference population under investigation?	Y	Y	Y	Y	N/A	Y	Y	Y	Y	U	Y	Y	Y	Y	U	Y	Y	Y	Y	Y
Were measures undertaken to address and categorise non-responders?	N	Y	Y	N	N/A	Y	Y	Y	Y	Y	N	Y	N	U	N	Y	Y	Y	N	N
Were the risk factor and outcome variables measured appropriate to the aims of the study?	N	Y	Y	Y	Y	Y	Y	Y	Y	Y	Y	Y	Y	Y	Y	Y	Y	Y	Y	Y
Were the risk factor and outcome variables measured correctly using instruments/measurements that had been trialled, piloted or published previously?	Y	Y	Y	Y	Y	Y	Y	Y	Y	Y	Y	Y	Y	Y	U	Y	Y	Y	U	U
Is it clear what was used to determined statistical significance and/or precision estimates? (e.g. p-values, confidence intervals)	Y	Y	N	Y	Y	Y	Y	Y	Y	N	Y	Y	N	Y	N	Y	Y	N	Y	Y
Were the methods (including statistical methods) sufficiently described to enable them to be repeated?	U	Y	N	Y	Y	Y	Y	Y	Y	N	Y	Y	N	Y	N	Y	Y	N	Y	Y
														Follow-up unclear, as mentioned in discussion	Method is brief					No headings, methodology unclear
Results																				
Were the basic data adequately described?	Y	Y	Y	Y	Y	Y	Y	Y	Y	Y	Y	Y	Y	Y	Y	Y	Y	Y	Y	Y
Does the response rate raise concerns about non-response bias?	N	N	N	N	N	N	N	N	N	N	N	N	N	N	N	N	N	Y	N	N
If appropriate, was information about non-responders described?	N/A	N/A	N/A	N/A	N/A	N/A	N/A	N/A	N/A	N/A	N/A	N/A	N/A	N/A	N/A	N/A	N/A	Y	N/A	N/A
Were the results internally consistent?	Y	Y	Y	Y	Y	Y	Y	Y	Y	Y	Y	Y	Y	Y	U	Y	Y	Y	Y	Y
Were the results presented for all the analyses described in the methods?	Y	Y	N	Y	Y	Y	Y	Y	Y	Y	Y	Y	Y	Y	U	Y	Y	U	Y	Y
															Although results provided, there appears to be no logical flow			Participant died		
Discussion																				
Were the authors' discussions and conclusions justified by the results?	Y	Y	Y	Y	Y	Y	Y	Y	Y	Y	Y	Y	Y	Y	Y	Y	Y	Y	Y	Y
Were the limitations of the study discussed?	N	N	Y	Y	Y	Y	Y	Y	Y	N	Y	Y	Y	N	N	Y	N	Y	Y	Y
													"Cons" were discussed in the methods							
Other																				
Were there any funding sources or conflicts of interest that may affect the authors' interpretation of the results?	N	N	Y	Y	Y	Y	Y	Y	Y	Y	Y	Y	Y	Y	N	Y	Y	N	Y	N
Was ethical approval or consent of participants attained?	N	N	Y	Y	Y	Y	Y	Y	Y	Y	Y	Y	Y	Y	N	Y	Y	N	Y	N
	Ethics not visible, possibly due to it being a retrospective study																			
Y - Yes: N - No: U - Uncertain; N/A - Not applicable																				
